# Laser-driven luminescent ceramic-converted near-infrared II light source for advanced imaging and detection techniques

**DOI:** 10.1038/s41377-025-01953-4

**Published:** 2025-09-11

**Authors:** Simin Gu, Huiwang Lian, Rongyi Kuang, Bibo Lou, Chonggeng Ma, Gaochao Liu, Jing Wang

**Affiliations:** 1 Ministry of Education Key Laboratory of Bioinorganic and Synthetic Chemistry, State Key Laboratory of Optoelectronic Materials and Technologies, School of Chemistry, Sun Yat-sen University, Guangzhou, 510006 China; 2 School of Optoelectronic Engineering & CQUPT-BUL Innovation Institute, Chongqing University of Posts and Telecommunications, Chongqing, 400065 China; 3 The State Key Laboratory of Luminescent Materials and Devices, Guangdong Provincial Key Laboratory of Fiber Laser Materials and Applied Techniques, School of Materials Science and Technology, South China University of Technology, Guangzhou, 510641 China; 4 Northeast Guangdong Key Laboratory of New Functional Materials, Guangdong Rare Earth Photofunctional Materials Engineering Technology Research Center, School of Chemistry and Environment, Jiaying University, Meizhou, 514015 China

**Keywords:** Lasers, LEDs and light sources, Optical materials and structures

## Abstract

Laser-driven near-infrared II (NIR-II) light sources comprising luminescent ceramics represent a promising research frontier, yet their development remains constrained by the external quantum efficiency (EQE) and thermal stability bottleneck of current luminescent materials. Herein, we present a non-equivalent cation substitution strategy to fabricate high-efficiency translucent MgO:Ni^2+^, Cr^3+^ NIR-II luminescent ceramics. The co-doping of Cr^3+^ induces structural distortion at Ni^2+^-occupied octahedral sites, effectively breaking the parity-forbidden d-d transition constraint while enabling efficient energy transfer from Cr^3+^ to Ni^2+^. These synergistic effects yield remarkable internal and external quantum efficiencies of 61.06% and 39.69%, respectively. The developed ceramic demonstrates exceptional thermal management capabilities with 31.28 W·m^−1^·K^−1^ thermal conductivity and 92.11% emission retention at 478 K. When integrated into laser-driven NIR-II light sources, the system achieves record-breaking performance of 214 mW output power under 21.43 W/mm^2^ blue laser excitation. Practical demonstrations showcase superior non-destructive imaging capabilities with 5.29 lp/mm spatial resolution and 0.97 contrast ratio. This work establishes a new paradigm for developing high-performance NIR-II light sources in advanced imaging and detection technologies.

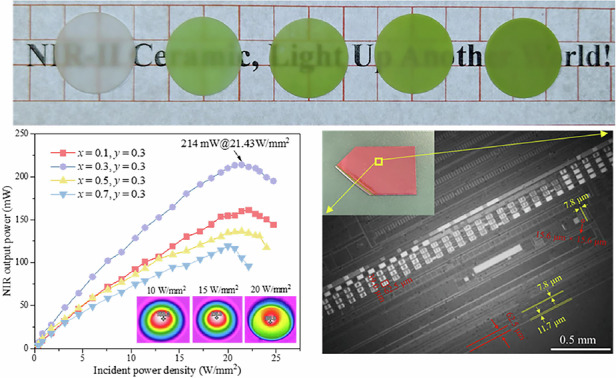

## Abstract (web summary)

**TOC**: Images of polished MgO:*x*%Ni^2+^, *y*%Cr^3+^ (*x* = 0-0.7, *y* = 0 and 0.3) ceramics under natural light and NIR-II output power of laser-driven NIR-II device based on MgO:*x*%Ni^2+^, 0.3%Cr^3+^ (*x* = 0.1-0.7) ceramics under different incident blue laser power densities. This work paves a new avenue for the future development of high-performance Ni^2+^-activated NIR-II emissive luminescence ceramics for laser-driven non-destructive real-time high-power NIR-II imaging and detection applications.

## Introduction

Near-infrared (NIR) light sources have revolutionized multiple fields including biomedical imaging, material analysis, and security screening through their non-destructive detection capabilities^1–4^. While traditional incandescent sources suffer from low efficiency and bulkiness, phosphor-converted LEDs (pc-LEDs) initially emerged as compact alternatives^5–7^. However, three fundamental limitations persist: inherent LED efficiency droop^8,9^, phosphor thermal degradation and poor thermal conductivity (~0.5 W·m^−1^·K^−1^) of organic binders^10,11^. These challenges have propelled the development of near-infrared luminescent ceramic-converted laser diodes (NIR lc-LDs), which combine laser diode excitation with ceramic converters offering superior thermal stability and high-power endurance^12–14^.

Current lc-LD research predominantly focuses on first biological window (NIR-I, 700–900 nm) emitters, particularly Cr^3+^-activated systems. Notable achievements include Y_2_CaA_l4_SiO_12_:Cr^3+^ ceramic showing ~680 mW at 184 W/cm^2^ (ref. ^15^), Gd_3_Al_2_Ga_3_O_12_:Cr^3+^ ceramic delivering 1.65 W output at 5.5 W excitation^16^ and MgO:Cr^3+^ ceramic reaching 6.36 W at 22 W/mm^2^ excitation^17^. Nevertheless, the second biological window (NIR-II, 900–1700 nm) offers superior performance for deep-tissue imaging and compositional analysis due to reduced scattering and enhanced molecular absorption^18–21^. This creates urgent demand for efficient NIR-II emitters compatible with high-power laser excitation.

Ni^2+^ ions with 3d^8^ electronic configuration have emerged as promising NIR-II activators in octahedral-site^22–24^. We firstly reported a MgO:Li^+^, Ni^2+^ phosphors achieving 19.7 mW output with emission wavelength ranging from 1330 to 1460 nm^25^. Unfortunately, the as-developed phosphor cannot be efficiently excited by the commercially used LED or LD blue chips. Thereafter, many optimized systems like MgGa_2_O_4_:Ni^2+^ were developed, exhibiting EQE of 29.4% with emissions at 1260 nm^26^ and 27.4 mW output at 350 mA^27^. However, two critical challenges persist: (1) The parity-forbidden nature of 3d-3d transitions of Ni^2+^ fundamentally limits external quantum efficiency; (2) The difficulty of converting the phosphor powder into ceramic, which could perfectly improve the thermal conductivity of Ni^2+^ ion activated luminescence materials to meet the need of high power NIR-II lc-LDs. Consequently, developing a Ni^2+^ ion activated NIR-II luminescent ceramics with higher external quantum efficiency and excellent thermal conductivity are in great and urgent need.

In this work, we successfully developed MgO:Ni^2+^, Cr^3+^ luminescent ceramics based on non-equivalent cation substitution strategy and high temperature ceramic sintering method. More importantly, non-equivalent cation substitution of Mg^2+^ by co-doping with Cr^3+^ results in a high lattice distortion in [NiO_6_] octahedral, which breaks the parity-forbidden 3d-3d transition of Ni^2+^ and consequently improves its luminescence efficiency. At the same time, resonant energy transfer from Cr^3+^ to Ni^2+^ further improves the luminescence efficiency of Ni^2+^. The resultant MgO:N^2+^, Cr^3+^ ceramics achieve unprecedented 39.69% EQE with NIR-II emissions at 1330 nm, high thermal conductivity of 31.28 W·m^–1^·K^–1^, and excellent anti-luminescence thermal quenching of 92.11%@478 K. Implemented in laser-driven devices, these ceramics enable record 214 mW output power under 21.43 W/mm^2^ blue laser excitation. Benefitting from the fabricated NIR-II lc-LDs based on translucent MgO:Ni^2+^, Cr^3+^ ceramics, we successfully demonstrated non-destructive imaging capabilities with spatial resolution of 5.29 lp/mm and 0.97 contrast ratio. This work provides rational design of Ni^2+^-activated NIR-II luminescent ceramics for next-generation laser-driven NIR-II lighting source, thereby meeting the growing demands in non-destructive detection and imaging applications.

## Results

**Fig. 1:**
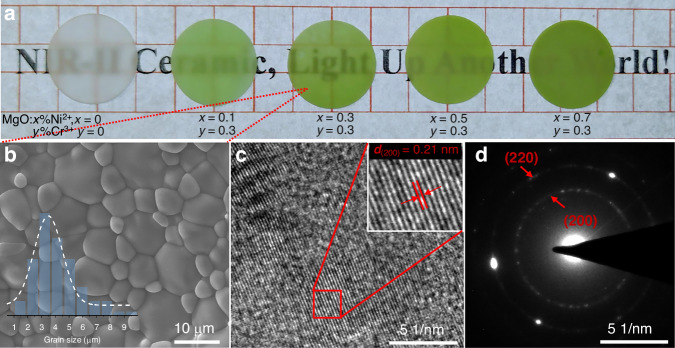
Morphology and structure of MgO:Ni^2^^+^,Cr^3^^+^ translucent ceramics. **a** Images of polished MgO:*x*%Ni^2+^, *y*%Cr^3+^ (*x* = 0–0.7, *y* = 0 and 0.3) ceramics under natural light. **b** The SEM image of MgO:0.3%Ni^2+^, 0.3%Cr^3+^ ceramic and the left bottom inset shows the histogram of grain size distribution. **c** The TEM image of MgO:0.3%Ni^2+^, 0.3%Cr^3+^ ceramic and the right top inset shows the enlarged part of lattice fringe marked in red square. **d** The SAED of MgO:0.3%Ni^2+^, 0.3%Cr^3+^ ceramic

Blank MgO ceramic and MgO:*x*%Ni^2+^, 0.3%Cr^3+^ (*x* = 0.1–0.7) translucent ceramics are listed in Fig. 1a from left to right, exhibiting color changes from translucency white to green-like. Clear grain particle and grain boundaries are observed in scanning electron microscope (SEM) images in Fig. 1b, confirming the successful synthesis of ceramic with high densification of 3.39 g/cm^3^ that reaches to 94.69% of the theoretical density of MgO (3.58 g/cm^3^). After carefully optimizing the doping concentration of Ni^2+^ and Cr^3+^ ions, which is one of crucial factors for the NIR-II emission performance, MgO:0.3%Ni^2+^, 0.3%Cr^3+^ ceramic was selected for detailed characterization. The grain size changes from 1 to 10 μm in the MgO:0.3%Ni^2+^, 0.3%Cr^3+^ ceramic and mainly grain size is concentrated in the 3–4 μm (see inset of Fig. 1b). The obtained MgO:0.3%Ni^2+^, 0.3%Cr^3+^ phosphor had serious agglomeration at the micron level (Supllementary Fig. S1). In contrast to the agglomeration in sintered phosphor, the fabricated ceramics show a characteristic grain structure with a flatter surface and no visible pores, which will contribute to the increased absorption of incident blue light, as discussed later. The transmission electron microscopy (TEM) images show an excellent crystallinity of MgO:0.3%Ni^2+^, 0.3%Cr^3+^ ceramic (Fig. 1c), where a clear lattice spacing of 0.21 nm is consistent with the (200) lattice plane of MgO. The selected area electron diffraction (SAED) in Fig. 1d corresponds to the (200) and (220) lattice planes of the MgO, also revealing the existence of pure MgO phase. Furthermore, X-ray diffraction (XRD) patterns (Supllementary Fig. S2a, b) confirm that all MgO:*x*%Ni^2+^, *y*%Cr^3+^ ceramics have a single MgO phase. No obvious new peaks and peak shift are observed in XRD patterns, suggesting a small amount of activator ions show no obvious influence on the MgO phase. The MgO host has a cube structure belonging to the *Fm-3m* space group with a uniform [MgO_6_] octahedral structure inside. The doped Ni^2+^ and Cr^3+^ ions were expected to replace Mg^2+^ cation in the octahedral site, due to the similar ionic radius of Ni^2+^ (0.69 Å), Cr^3+^ (0.62 Å) and Mg^2+^ (0.72 Å)^28^.

**Fig. 2:**
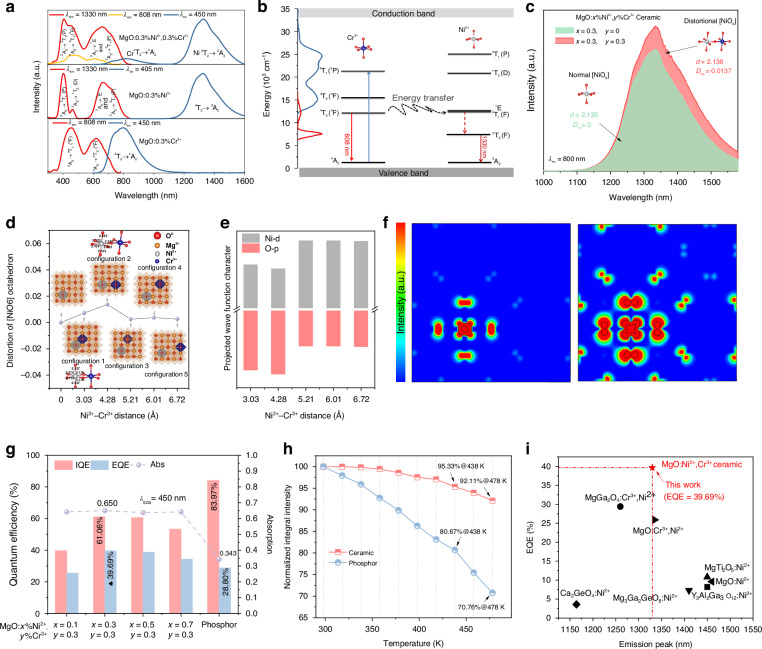
Luminescence properties of MgO:Ni^2^^+^,Cr^3+^ translucent ceramics. **a** PLE and PL spectra of MgO:0.3%Ni^2+^, 0.3%Cr^3+^, MgO:0.3%Ni^2+^, and MgO:0.3%Cr^3+^ ceramics. **b** Energy level diagram of Cr^3+^/Ni^2+^ in MgO:0.3%Ni^2+^, 0.3%Cr^3+^ ceramic and the mechanism of energy transfer from Cr^3+^ to Ni^2+^. **c** Comparison of NIR-II PL spectra of Ni^2+^ in octahedron cation site with different distortion degrees in MgO:0.3%Ni^2+^ and MgO:0.3%Ni^2+^, 0.3%Cr^3+^ ceramics. **d** The distortion of [NiO_6_] octahedron in MgO:Ni^2+^ and MgO:Ni^2+^, Cr^3+^ with different Ni^2+^-Cr^3+^ distances. Insets show local structure of [NiO_6_] octahedron in MgO:Ni^2+^ and MgO:Ni^2+^, Cr^3+^ with Ni^2+^-Cr^3+^ distances of 3.03, 4.28, 5.21, 6.01 and 6.72 Å, respectively. **e** Contributions of the O-2*p* and Ni-3*d* orbitals to the highest occupied 3*d* Kohn–Sham orbitals as a function of Ni^2+^-Cr^3+^ separation (3.03, 4.28, 5.21, 6.01 and 6.72 Å). **f** Band-decomposed charge density profiles for a single Ni^2+^ dopant (left) and Ni^2+^-Cr^3+^ dopants separated by 4.28 Å (right). These profiles represent the Ni^2+^ 3d^8^ Kohn–Sham orbitals within the (0 6 0) Miller plane, with a saturation scale from 0.0001 (blue) to 0.002 (red) and linear contour intervals from 0.01 to 0.3, in units of *a*-3 0, where *a*_0_ is the Bohr radius. **g** Absorption (dash-dot line), IQE (red column) and EQE (blue column) of MgO:*x*%Ni^2+^, 0.3%Cr^3+^ (*x* = 0.1–0.7) ceramics and MgO:0.3%Ni^2+^, 0.3%Cr^3+^ phosphor. **h** The trend of integrated emission intensities of MgO:0.3%Ni^2+^, 0.3%Cr^3+^ ceramic and phosphor dependent on temperatures. **i** Comparison on EQE values of MgO:0.3%Ni^2+^, 0.3%Cr^3+^ ceramic and previously reported NIR-II emissive Ni^2+^-activated phosphors

Figure 2a shows the photoluminescence (PL) and PL excitation (PLE) spectra of MgO:0.3%Ni^2+^, 0.3%Cr^3+^, MgO:0.3%Ni^2+^, and MgO:0.3%Cr^3+^ ceramics. The MgO:0.3%Cr^3+^ ceramic and MgO:0.3%Ni^2+^ ceramic show a single emission peaking at 808 or 1330 nm, corresponding to the ^4^T_2_ → ^4^A_2_ transition of Cr^3+^ and the ^3^T_2_ → ^3^A_2_ transition of Ni^2+^, respectively. The PLE spectra of MgO:0.3%Cr^3+^ and MgO:0.3%Ni^2+^ ceramics are different from each other. The characteristic excitation peaks of MgO:0.3%Cr^3+^ ceramic are mainly located at 450 and 620 nm, due to the ^4^A_2_ → ^4^T_1_ (^4^F) and ^4^A_2_ → ^4^T_2_ (^4^F) transitions of Cr^3+^ (ref. ^17^) and that of MgO:0.3%Ni^2+^ ceramic are located at 405, 460 and 660 nm, arising from the ^3^A_2_ → ^3^T_1_ (P), ^3^A_2_ → ^1^T_2_ (D), ^3^A_2_ → ^1^E and ^3^A_2_ → ^3^T_1_ (F) transitions of Ni^2+^ (ref. ^29^). For MgO:0.3%Ni^2+^, 0.3%Cr^3+^ ceramic, the PLE spectra mainly consist of characteristic excitation peaks of Cr^3+^ and Ni^2+^ ions when monitored at 1330 nm from Ni^2+^ NIR-II emission. The PL spectra exhibit a predominant band at 1330 nm and a weak shoulder band at 808 nm, associated with Ni^2+^ (ref. ^25^) and Cr^3+^ (ref. ^17^) ions, respectively, when excited at 450 nm that from one of the characteristic excitation peaks of Cr^3+^. The PLE spectra of MgO:0.3%Ni^2+^, 0.3%Cr^3+^ match well with the blue emission of the commercial blue laser chip, which makes it more easily and efficiently pumped by commercial laser chip for laser-driven NIR-II lighting source application. The overlap between the PLE spectrum of MgO:0.3%Ni^2+^ and the PL spectrum of MgO:0.3%Cr^3+^ suggests the possible occurrence of efficient resonant energy transfer (ET) from Cr^3+^ to Ni^2+^ in MgO:0.3%Ni^2+^, 0.3%Cr^3+^. Moreover, both the dependence of the NIR-II emission of Ni^2+^ ions on the concentration of Cr^3+^ ions (Supllementary Fig. S3) and the dependence of the lifetime of Cr^3+^ ions on the concentration of Ni^2+^ ions (Supllementary Fig. S4, Supllementary Table S1 and the lifetime were calculated by Supllementary Eq. (S2)) also strongly support the appearance of efficient resonant ET from Cr^3+^ to Ni^2+^ (refs. ^30–32^). The mechanism diagram of ET from Cr^3+^ to Ni^2+^ is proposed (Fig. 2b) and the ET efficiency^26^ (${\eta }_{{ET}}$) was calculated by Supllementary Eq. (S3). The highest ${\eta }_{{ET}}$ is 60.16% in the MgO:0.3%Ni^2+^, 0.3%Cr^3+^ ceramic.

Besides efficient resonant energy transfer discussed above, the distortion degree of [NiO_6_] induced by non-equivalent co-doping Cr^3+^ ions also plays an important role in enhancing the NIR-II emission of Ni^2+^. It is well known that *3d-3d* transitions of Ni^2+^ ions at regular octahedral sites is parity-forbidden due to their intra-configurational nature, usually resulting in low excitation efficiency that will consequently lead to poor emission intensity. In MgO, Ni^2+^ ions occupy the ideal regular [MgO_6_] octahedron site with a distortion index of 0, which indeed leads to poor NIR-II emission (see green curve in Fig. 2c). In theory, a highly distorted local coordination environment, particularly one lacking inversion symmetry, is often associated with strong odd-parity crystal field potentials that can relax parity-forbidden restriction by mixing the p states, such as O-2p states, into the *3d* states of the transition metal ions and will increase the *3d-3d* absorption probabilities and enhance *3d-3d* emission intensities of Ni^2+^ ions. Hereafter, we will evaluate in detail the distortions of [NiO_6_] octahedra induced by non-equivalent co-doping with Cr^3+^ ions. In MgO:Ni^2+^, Cr^3+^, five possible rearrangement configurations of Ni^2+^-Cr^3+^ ion pairs at various interionic distances are taken into account, which will generates different chemical stress within the local coordination environment of Ni^2+^ ion. First-principles geometry optimization reveals that the distortion index of the [NiO_6_] octahedron, as defined by Baur^33^, is larger in the Ni^2+^/Cr^3+^ co-doped case than in the Ni^2+^ singly-doped case (Fig. 2d and Supllementary Table S2). For both nearest- and next-nearest-neighbor Cr^3+^ arrangements around central Ni^2+^ ions (configuration 1 and 2 in Fig. 2d), the distortions of the [NiO_6_] octahedron are more serious, with the distortion index of 0.7% for nearest-case and 1.4% for next-nearest-case, respectively. The difference in distortion index between these two cases arises because, in the nearest-neighbor configuration, Cr^3+^ co-doping creates an isotropic effect, sharing two oxygen ligands with the central Ni^2+^ ion in the *x*-*y* plane and in contrast, in the next-nearest configuration, only one oxygen ligand links the Cr^3+^ and Ni^2+^ ions along the *x* direction, resulting in a larger distortion index. Furthermore, first-principles electronic structure calculations evaluated the hybridization between the 3*d* states of central Ni^2+^ ions and the 2*p* states of their nearest O^2-^ ions (Fig. 2e). The results demonstrate that as the co-doped Cr^3+^ ions are progressively closer to the central Ni^2+^ ions, the contribution of the central Ni-3*d* states to the highest occupied 3*d* Kohn–Sham orbitals decreases while the contribution from the nearest oxygen ligands’ 2*p* states increases. The charge density profiles of the central Ni^2+^ ion and its nearest oxygen ligands (Fig. 2f) further confirm this rearrangement of the electron cloud and resulting hybridization. These findings strongly indicate that local distortions in the ligand environment generate strong odd-parity crystal field potentials, significantly mixing the O-2*p* states with the Ni-3*d* states and strongly breaking the parity-forbidden nature of 3*d*-3*d* transitions, which will enhance *3d-3d* emission intensities of Ni^2+^ ions. Indeed, it is obviously seen in Fig. 2c that compared to MgO:0.3%Ni^2+^, the NIR-II emission intensity of Ni^2+^ ions in MgO:0.3%Ni^2+^, 0.3%Cr^3+^ is enhanced by about 1.33 times when directly monitored the *3d-3d* excited transition of Ni^2+^ ion located at 800 nm that cannot excite Cr^3+^ ions. Based on the above theoretical and experimental discussions, we conclude that non-equivalent co-doping of Cr^3+^ ions successfully breaks the *3d-3d* parity-forbidden nature of Ni^2+^ and enhances the NIR-II emission of Ni^2+^ ions.

Benefited from breaking of the *3d-3d* parity forbiddance of Ni^2+^ ion in octahedral sites and efficient resonant energy transfer, the MgO:0.3%Ni^2+^, 0.3%Cr^3+^ ceramic shows high IQE and EQE of 61.06% and 39.69%, respectively, when the NIR-II emission band focused on 1000-1650 nm under 450 nm excitation (Fig. 2g, Supllementary Fig. S5, Supllementary Table S3 and Supllementary Eq. (S4)). Meanwhile, the NIR-II IQE and EQE values of MgO:0.3%Ni^2+^, 0.3%Cr^3+^ reference phosphor are 83.97% and 28.80% (Supllementary Fig. S6 and Supllementary Table S3), respectively. Comparatively, MgO:0.3%Ni^2+^, 0.3%Cr^3+^ ceramic with the same chemical composition have a relatively lower IQE but a higher EQE. This is mainly because ceramics have a better compact and uniform grain arrangement than phosphor, which induces the increase in absorption of incident blue excitation light from 34.3% of MgO:0.3%Ni^2+^, 0.3%Cr^3+^ phosphor up to 65.0% of MgO:0.3%Ni^2+^, 0.3%Cr^3+^ ceramic (Fig. 2g).

In general, luminescence will partly or totally quench if the luminescence material is used at higher temperature, especially for high-power laser-driven application scenarios with high thermal effect. This phenomenon is the so-called luminescence thermal quenching behavior. The temperature-dependent PL spectra of MgO:0.3%Ni^2+^, 0.3%Cr^3+^ ceramic and MgO:0.3%Ni^2+^, 0.3%Cr^3+^ phosphor were comparatively measured (Supllementary Fig. S7a, b). It is found the MgO:0.3%Ni^2+^, 0.3%Cr^3+^ ceramic shows more excellent anti-luminescence thermal quenching behavior than the MgO:0.3%Ni^2+^, 0.3%Cr^3+^ phosphor. Compared to the initial integral emission intensity at room temperature, the ceramic maintains 92.11% at 478 K, much higher than that of phosphor (70.76%) (Fig. 2h). This is mainly due to the advantages of dense and uniform grain arrangements and high phase purity degree of ceramic, compared to phosphor, which can efficiently conduct the ambient heat away. The thermal conductivity of MgO:0.3%Ni^2+^, 0.3%Cr^3+^ ceramic was estimated to be 31.28 W·m^−1^·K^−1^ (Supllementary Fig. S8 and Supllementary Eq. S5), strongly supporting the excellent anti-luminescence thermal quenching behavior of MgO:0.3%Ni^2+^, 0.3%Cr^3+^ ceramic. Besides the excellent anti-luminescence thermal quenching behavior, MgO:0.3%Ni^2+^, 0.3%Cr^3+^ ceramic exhibits more excellent anti-thermal drifting of the emission peak and anti-thermal broadening of the full width of half maximum (FWHM). As the temperature increases from 298 to 478 K, the emission peak of Ni^2+^ ion shows a tiny redshift from 1325 to 1340 nm and its FWHM broadens slightly from 198 to 221 nm (Supllementary Fig. S7c, d). In summary, all the above properties of MgO:Ni^2+^, Cr^3+^ ceramic including well matching between its excitation band and the emission of the commercial blue laser chip, high EQE, excellent anti-luminescence quenching behavior, excellent anti-thermal drifting of the emission peak and anti-thermal broadening of the FWHM and high thermal conductivity, strongly suggest MgO:Ni^2+^, Cr^3+^ ceramic is promising for high-performance laser-driven NIR-II light sources.

**Fig. 3:**
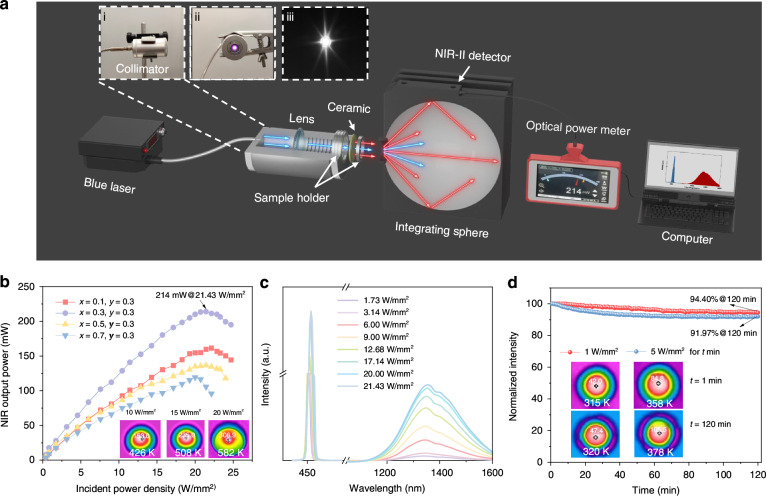
Laser-driven NIR-II device and luminescence properties. **a** The schematic diagram of the laser-driven NIR-II device and NIR optical measurement system. The insets are photographs of (i) the collimator, images of the working NIR-II lighting source taken by (ii) visible camera and (iii) NIR-II camera. **b** NIR-II output power of laser-driven NIR-II device based on MgO:*x*%Ni^2+^, 0.3%Cr^3+^ (*x* = 0.1–0.7) ceramics under different incident blue laser power densities. The inserts are thermographs of the irradiation spot of MgO:0.3%Ni^2+^, 0.3%Cr^3+^ceramic at 10, 15 and 20 W/mm^2^ blue laser excitation, respectively. **c** Input-power-density dependent PL spectra of MgO:0.3%Ni^2+^, 0.3%Cr^3+^ ceramic. **d** Luminescence stability of MgO:0.3%Ni^2+^, 0.3%Cr^3+^ ceramic under continuous 1 and 5 W/mm^2^ blue laser excitation for 120 min. The inserts are thermographs of the irradiation spot of this ceramic at 1 and 5 W/mm^2^ blue laser excitation for 1 and 120 min, respectively

Figure 3a shows an actual photograph and a schematic diagram of the laser-driven NIR-II lighting source. The blue laser beam focused by the optical fiber enters the collimator and is converted into parallel blue laser lights by the convex lens, then enters the MgO:0.3%Ni^2+^, 0.3%Cr^3+^ ceramic, and finally broadband NIR-II light is generated and transmitted through the MgO:0.3%Ni^2+^, 0.3%Cr^3+^ ceramic. The optical properties of the laser-driven NIR-II lighting source were recorded by a NIR optical measurement system including integrating sphere with NIR-II detector and optical power meter.

The dependence of NIR-II output power of the as-fabricated laser-driven NIR-II lighting source based on MgO:x%Ni^2+^, 0.3%Cr^3+^ ceramic (*x* = 0.1–0.7) on the incident blue laser power densities are shown in Fig. 3b. Under exposure on the high-energy laser, blank MgO ceramic will generate heat and lead to a false output power of the laser-driven device based on blank MgO ceramic even though blank MgO ceramic does not have NIR emission. This will cause a test deviation on MgO:Ni^2+^, Cr^3+^ ceramic. Therefore, the accurate output power of laser-driven device based on MgO:Ni^2+^, Cr^3+^ ceramic was obtained after subtracting the output power of laser-driven device based on blank MgO ceramic (Supllementary Fig. S9). For different MgO:Ni^2+^, Cr^3+^ ceramics, the NIR-II output power of all the devices gradually increases, then reaches a saturation point and finally drops. And among the MgO:x%Ni^2+^, 0.3%Cr^3+^ (*x* = 0.1–0.7), MgO:0.3%Ni^2+^, 0.3%Cr^3+^ ceramic always shows the highest NIR-II output power at the same incident power density in the whole range of 0–25 W/mm^2^, mainly due to its highest EQE as supported by Fig. 2d. For MgO:0.3%Ni^2+^, 0.3%Cr^3+^ ceramic, its saturation point comes with the incident power density of 21.43 W/mm^2^. Before this point, the reason for the nearly linear increase of the NIR-II output power is that the increased optical power density of incident blue laser makes more activators Cr/Ni excited and consequently contributes to enhanced NIR-II emission (Fig. 3b, c). After this point, the heat on the ceramic becomes seriously accumulated as the optical power density of the incident blue laser increases, which will lead to obvious luminescence thermal quenching of MgO:0.3%Ni^2+^, 0.3%Cr^3+^ ceramic that counteracts the NIR-II emission enhancement caused by the increase of the excitation power density. The thermographs (inset of Fig. 3b) of MgO:0.3%Ni^2+^, 0.3%Cr^3+^ ceramic under blue-laser excitation with different power densities indeed show that the target temperature of ceramic increases from 426 to 508 and 582 K at incident power densities of 10, 15 and 20 W/mm^2^. Taking the luminescence thermal quenching trend of MgO:0.3%Ni^2+^, 0.3%Cr^3+^ ceramic (Fig. 2e) into account, the thermal quenching behavior under high incident power density is mainly the reason for the decline of the NIR-II output power of the laser-driven NIR-II lighting source based on MgO:0.3%Ni^2+^, 0.3%Cr^3+^ ceramic. Most importantly, laser-driven NIR-II lighting source based on MgO:0.3%Ni^2+^, 0.3%Cr^3+^ ceramic exhibits NIR-II output power of 214 mW under incident blue laser power density of 21.43 W/mm^2^. Till now, it is the highest recorded NIR-II output power, compared to the previously reported works (Supllementary Table S3). Moreover, the time-dependent luminescence declines of the as-fabricated laser-driven NIR-II lighting source based on MgO:0.3%Ni^2+^, 0.3%Cr^3+^ ceramic at fixed incident power density of 1 and 5 W/mm^2^ was recorded in Fig. 3d. The luminescence performance of device at whatever 1 or 5 W/mm^2^ gradually declines as the working time increases. Apparently, the decline trend at 1 W/mm^2^ is smaller than at 5 W/mm^2^. The device at continuous incident power density of 1 and 5 W/mm^2^ for 120 mins keeps 94.40% and 91.97% of initial integral NIR-II emission intensity for 1 min, respectively. The difference is mainly because after continuous blue laser excitation with high incident power density for 120 minutes, the temperature of ceramic is 378 K higher than 320 K at low continuous incident power density (insert of Fig. 3d) and consequently higher heat effect leads to relatively more serious luminescence quenching behavior.

For comparison with the laser-driven NIR-II lighting source, LED-driven devices based on MgO:*x*%Ni^2+^, 0.3%Cr^3+^ (*x* = 0.1–0.7) ceramics (Supllementary Fig. S10) were also fabricated. The 450 nm blue LED chip shows the highest blue optical power of 0.96 W at 700 mA current and thereafter blue optical power decreases (Supllementary Fig. S10a). The LED-driven NIR-II lighting source based on MgO:0.3%Ni^2+^, 0.3%Cr^3+^ gives the highest NIR-II output power of 93.5 mW at incident current of 600 mA (Supllementary Fig. S10b), which is the highest record among current and previous LED-driven NIR-II lighting sources but only about 44% (214 mW@ 21.43 W/mm^2^) of laser-driven NIR-II lighting source based on the same ceramic (Supllementary Table S4).

**Fig. 4:**
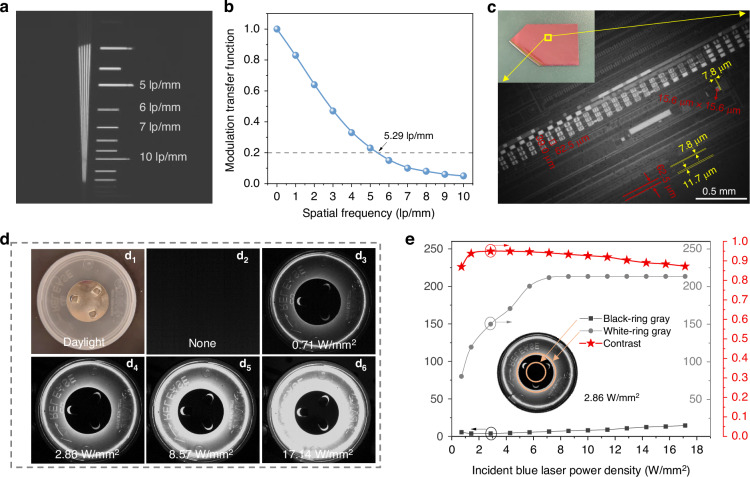
Applications based on laser-driven NIR-II light sources. **a** Spatial resolution of our laser-driven NIR-II light sources based on MgO:0.3%Ni^2+^, 0.3%Cr^3+^ ceramic, determined by a standard line-pair card (lp/mm). **b** Modulation transfer function curves of our laser-driven NIR-II light sources based on MgO:0.3%Ni^2+^, 0.3%Cr^3+^ ceramic. **c** The photographs of a commercial silicon wafer with internal integrated micro-structure taken by a NIR camera under the obtained NIR-II light source and a visible camera under daylight (see the top left inset). **d** The photographs of an iron sheet on a plastic box: taken by a visible camera under daylight (d_1_), and by a NIR camera in the dark (d_2_) and (d_3_–d_6_) under exposure to our NIR-II lighting source at different blue laser power densities. **e** The grayscale variation trend of the NIR images of white-ring (solid dot line) of the plastic box and black-ring (solid square line) of the iron sheet and the contrast variation trend (solid star line) of the white/black ring NIR images, under exposure to our NIR-II lighting source at different blue laser power densities. Inset is the NIR image of the iron sheet on a plastic box under 2.86 W/mm^2^ excitation

Based on the superior performances of the as-fabricated device above, it is convinced that MgO:0.3%Ni^2+^, 0.3%Cr^3+^ ceramic shows promising potential for non-invasive real-time high-power NIR imaging and detection applications. Generally, spatial resolution and contrast are two key factors for practical non-invasive real-time high-power NIR imaging and detection. First, a standard line-pair card was utilized to evaluate the spatial resolution ability of laser-driven NIR-II lighting source based on MgO:Ni^2+^, Cr^3+^ ceramic for NIR imaging. Impressively, an excellent spatial resolution of 5.29 lp/mm is achieved (Fig. 4a, b), demonstrating the great potential of our laser-driven NIR-II lighting source for non-invasive real-time discrimination of closely spaced lines or contours on target objects. The inset of Fig. 4c shows the photograph of a commercial silicon wafer taken by a visible camera in daylight. Obviously, one cannot see any internal microstructure. In contrast, under the exposure to our laser-driven NIR-II lighting source, the internal integrated micro-structure inside the commercial silicon wafer was captured clearly by the NIR camera and it is invisible to the naked eye (Fig. 4c). For instance, a metal bonding pad with the length of 62.5 μm and the width of 39.0 μm, a square wire structure with a length of 15.6 μm, and even wires with spacing distance of 7.8 μm are observed clearly. Second, the NIR imaging contrast was evaluated. Fig. 4d_1_ shows the photograph of one round iron sheet on a plastic box taken by a visible camera in daylight. The round iron sheet on the plastic box is invisible to NIR camera in the dark (Fig. 4d_2_) but is visible to NIR camera under exposure to our laser-driven NIR-II lighting source (Fig. 4d_3_–d_6_). A schematic diagram of capturing NIR images is shown in Supllementary Fig. S11. As the output power of the laser-driven NIR-II lighting source increases, due to the increase of the blue laser power density, both the circular iron sheet and the plastic box and even the letters “RELEASE” become clearer and clearer. The grayscale variation trend related to the incident blue laser power density (Fig. 4e) shows that the average gray of white-ring of the plastic box is 80.69 at 0.71 W/mm^2^, then sharply increases to 213.36 at 8.57 W/mm^2^ and finally increases slightly to 213.50 at 17.14 W/mm^2^ (solid dot line in Fig. 4e) while the average gray of black-ring of the iron sheet is 5.63 at 0.71 W/mm^2^, then decreases slightly to 3.83 at 1.43 W/mm^2^ and gradually increases to 14.56 at 17.14 W/mm^2^ (solid square line in Fig. 4e). Based on the above results, the contrast (solid star line in Fig. 4e) of these NIR images is estimated by Weber Contrast Equation^34,35^ (Supllementary Eq. (S6)). It is found that the highest contrast is 0.97 at 2.86 W/mm^2^ and the lowest contrast is 0.93 at 0.71 W/mm^2^. The excellent spatial resolution and superior contrast discussed above suggest the as-fabricated laser-driven NIR-II lighting source based on MgO:0.3%Ni^2+^, 0.3%Cr^3+^ ceramic shows great potential applications in the field of practical non-invasive real-time high-power NIR imaging and detection.

## Discussion

In this work, NIR-II emissive MgO:Ni^2+^, Cr^3+^ ceramics with record EQE of 39.69% and superior thermal conductivity of 31.28 W·m^−1^·K^−1^ have been successfully developed. Benefiting from the non-equivalent co-doping strategy in MgO:Ni^2+^, Cr^3+^, it helps to break the d-d parity forbidden nature of Ni^2+^ ion at octahedral sites and produce efficient resonant energy transfer from Cr^3+^ to Ni^2+^, which greatly contribute high EQE and remarkable thermal quenching of 92.11%@478 K. The as-fabricated laser-driven NIR-II light source shows a record broadband NIR-II output power of 214 mW under an incident blue laser power density of 21.43 W/mm^2^. Finally, we demonstrate its potential application in non-destructive real-time high-power NIR-II imaging and detection with an excellent spatial resolution of 5.29 lp/mm and superior contrast of 0.97. This work paves a new avenue for the future development of high-performance Ni^2+^-activated NIR-II emissive luminescence ceramics for laser-driven non-destructive real-time high-power NIR-II imaging and detection applications.

## Materials and methods

### Ceramic preparation

The MgO:*x*%Ni^2+^, *y*%Cr^3+^ (*x* = 0.1–0.7, *y* = 0.1–0.7) NIR-II emissive luminescence ceramics were fabricated by cool isostatic pressing and high-temperature sintering. The raw materials of MgO (99.99%, Aladdin), NiO (99.99%, Aladdin), and Cr_2_O_3_ (99.99%, Aladdin) were used directly without further purifications. They were weighted accurately according to the stoichiometric composition of the ceramics. The raw materials were mixed up and ball-milled for 12 h in an agate jar with ethanol as a dispersant. Then, the mixed powder was dried at 70 °C for 24 h in the oven and calcined at 600 °C for 2 h in the muffle furnace to remove volatile impurities. The calcined precursor powder was pressed into disks with a diameter of 17 mm and then further compacted by cold-isostatic-pressing (CIP) at 250 MPa for 15 min to obtain a green body. The green body was sintered at 1450 °C for 8 h in the air. Finally, the obtained ceramics were double-surface polished to 0.2 mm for further measurements. Two sets of samples were MgO:*x*%Ni^2+^, 0.3%Cr^3+^ (*x* = 0.1, 0.3, 0.5, 0.7) ceramics and MgO:0.5%Ni^2+^, *y*%Cr^3+^ (*y* = 0.1, 0.3, 0.5, 0.7) ceramics. At the same time, MgO:0.3%Ni^2+^, 0.3%Cr^3+^ NIR-II emissive phosphor was also considered to be a contrast sample, and it was synthesized in the same conditions only without CIP.

### Characterization

The X-ray diffraction (XRD) patterns were characterized using a diffractometer (Rigaku, D/MAX 2200 VPC) equipped with Cu Kα radiation (λ = 1.5405 Å). The scanning electron microscopy (SEM) of the ceramics was measured with a field-emission scanning electron microscope (ZEISS, Gemini SEM 500). The transmission electron microscope (TEM) and selected area electron diffraction (SAED) were acquired using a transmission electron microscope (Thermo Fisher Scientific, FEI TALOS-F200S). The photoluminescence (PL) and photoluminescence excitation (PLE) spectra, and luminescence decay curves were recorded on a high-resolution spectrofluorometer (Edinburgh Instruments, FLS1000) equipped with a 450 W xenon lamp. An absolute photoluminescence quantum yield spectrometer was employed to measure the quantum efficiency (Hamamatsu, C13534). The temperature-dependent spectra were evaluated by the FLS1000 spectrophotometer equipped with a MercuryiTc temperature control instrument (OXFORD). The NIR-II output power of the as-fabricated laser-driven NIR-II devices was measured by an integrating sphere sensor system (Thorlabs, S145C and PM400). The thermographs of laser-irradiated samples were measured by thermal imager (China, FOTRIC Inc. FOTRIC 225S). Visible photos and NIR images were taken by a visible camera (HUAWEI Mate50 pro) and a NIR camera (LD-SW6401715-CTE2-G, Xi’an Leading Optoelectronic Technology), respectively.

### Computational methods

All first-principles calculations were performed within the density functional theory (DFT) framework using the Vienna Ab initio Simulation Package (VASP)^36,37^. A 3 × 3 × 3 MgO supercell, containing 216 atoms, was employed to model both the Ni^2+^ singly-doped and Ni^2+^/Cr^3+^ co-doped systems. Spin-polarized DFT calculations were utilized due to the open-shell characteristics of the electronic configurations of Ni^2+^ and Cr^3+^ ions. Structural relaxations were carried out with the Perdew-Burke-Ernzerhof (PBE) functional^38^, incorporating a Hubbard *U*_eff_ parameter of 4 eV to account for the 3*d* electron correlations. After relaxation, the hybrid PBE0 functional^39^ was used to achieve a more accurate description of the electronic structure. Projector augmented wave pseudopotentials were applied to describe atomic interactions, with the valence-electron configurations of Ni, Cr, Mg, and O set as 3*p*^6^3*d*^9^4*s*^1^, 3*p*^6^3*d*^5^4*s*^1^, 2p^6^3*s*^2^, and 2*s*^2^2*p*^4^, respectively^40,41^. Brillouin zone sampling was performed using a single *Γ*-point. The plane-wave basis cutoff energy was set to 520 eV, with convergence criteria of 10^−5 ^eV for electronic energy minimization and 0.02 eV/Å for Hellmann–Feynman forces on each atom.

## Supplementary information

Suppoting information

## Data Availability

The datasets generated during and/or analyzed during the current study are available from the corresponding author on reasonable request.

## References

[1] Sun, Y. et al. Bright and stable perovskite light-emitting diodes in the near-infrared range. *Nature* **615**, 830–835 (2023). 36922588 10.1038/s41586-023-05792-4

[2] Saif, M. et al. Non-invasive monitoring of chronic liver disease via near-infrared and shortwave-infrared imaging of endogenous lipofuscin. *Nat. Biomed. Eng.* **4**, 801–813 (2020). 32572196 10.1038/s41551-020-0569-y PMC8310386

[3] He, Y. et al. Recent progress of nondestructive techniques for fruits damage inspection: a review. *Crit. Rev. Food Sci. Nutr.* **62**, 5476–5494 (2021). 33583246 10.1080/10408398.2021.1885342

[4] Vasilopoulou, M. et al. Advances in solution-processed near-infrared light-emitting diodes. *Nat. Photonics* **15**, 656–669 (2021).

[5] Qiao, J. et al. Divalent europium-doped near-infrared-emitting phosphor for light-emitting diodes. *Nat. commun.* **10**, 5267 (2019). 31748595 10.1038/s41467-019-13293-0 PMC6868216

[6] Jia, Z. et al. Strategies to approach high performance in Cr^3+^-doped phosphors for high-power NIR-LED light sources. *Light Sci. Appl.* **9**, 86 (2020). 32435469 10.1038/s41377-020-0326-8 PMC7229223

[7] Wang, S. et al. Achieving high quantum efficiency broadband NIR Mg4Ta2O9:Cr3+ phosphor through lithium ion compensation. *Adv. Mater* **35**, e2300124 (2023). 36867871 10.1002/adma.202300124

[8] Laubsch, A. et al. Indirect Auger recombination as a cause of efficiency droop in nitride light-emitting diodes. *Phys. Status Solidi C.* **6**, S913 (2009).

[9] Schubert, M. F. et al. Auger recombination in InGaN measured by photoluminescence. *Appl. Phys. Lett.* **91**, 141101 (2007).

[10] Miao, S. et al. Blue LED‐Pumped broadband short‐wave infrared emitter based on LiMgPO_4_:Cr^3+^, Ni^2+^ phosphor. *Adv. Mater. Technol.* **7**, 2200320 (2022).

[11] Tang, C. et al. Ni^2+^-activated MgTi_2_O_5_ with broadband emission beyond 1200 nm for NIR-II light source applications. *J. Mater. Chem. C.* **10**, 18234–18240 (2022).

[12] Wierer, J. et al. Comparison between blue lasers and light-emitting diodes for future solid-state lighting. *Laser Photonics Rev.* **7**, 963–993 (2013).

[13] Rahman, F. Diode laser-excited phosphor-converted light sources: a review. *Opt. Eng.* **61**, 060901 (2022).

[14] Kim, M. et al. Origin of efficiency droop in GaN-based light-emitting diodes. *Appl. Phys. Lett.* **91**, 183507 (2007).

[15] Zheng, G. et al. Glass-crystallized luminescence translucent ceramics toward high-performance broadband NIR LEDs. *Adv. Sci.* **9**, 2105713 (2022). 10.1002/advs.202105713 PMC8922114 35072364

[16] Jiang, H. et al. Ultra‐efficient GAGG:Cr^3+^ ceramic phosphor‐converted laser diode: a promising high‐power compact near‐infrared light source enabling clear imaging. *Adv. Optical Mater.* **10**, 2102741 (2022).

[17] Liu, G. et al. Laser-driven broadband near-infrared light source with watt-level output. *Nat. Photonics* **18**, 562 (2024).

[18] Lu, X. et al. Long-wavelength near-infrared divalent nickel-activated double-perovskite Ba_2_MgWO_6_ phosphor as imaging for human fingers. *ACS Appl. Mater. Interfaces* **15**, 39472–39479 (2023). 37552864 10.1021/acsami.3c04335

[19] Guo, X. et al. Valence state control of Cr^4+^-activated Li_2_SrGeO_4_ for NIR-II light source to distinguish deuterium and non-deuterium reagents. *J. Mater. Chem. C.* **11**, 7611–7618 (2023).

[20] Feng, Z. et al. Perfecting and extending the near-infrared imaging window. *Light Sci. Appl.* **10**, 197 (2021). 34561416 10.1038/s41377-021-00628-0 PMC8463572

[21] Siesler, H. W. et al. *Near-infrared spectroscopy: principles, instruments, applications* (John Wiley & Sons, Press, 2008).

[22] Rajendran, V. et al. Unraveling luminescent energy transfer pathways: futuristic approach of miniature shortwave infrared light-emitting diode design. *ACS Energy Lett.* **8**, 2395–2400 (2023).

[23] Brik, M. G. et al. Spectroscopic properties of Ni^2+^ ions in octahedral complexes. *Chin. J. Lumin.* **43**, 1459–1467 (2022).

[24] Yuan, L. et al. Ni^2+^-doped garnet solid-solution phosphor-converted broadband shortwave infrared light-emitting diodes toward spectroscopy application. *ACS Appl. Mater. Interfaces* **14**, 4265–4275 (2022). 35025207 10.1021/acsami.1c20084

[25] Liu, B. M. et al. Ultra-broadband and high-efficiency phosphors to brighten NIR-II light source applications. *Cell Rep. Phys. Sci.* **3**, 101078 (2022).

[26] Miao, S. et al. Broadband short-wave infrared-emitting MgGa_2_O_4_:Cr^3+^, Ni^2+^ phosphor with near-unity internal quantum efficiency and high thermal stability for light-emitting diode applications. *ACS Appl. Mater. Interfaces* **15**, 32580–32588 (2023). 37384930 10.1021/acsami.3c05980

[27] Liu, B. M. et al. A High-efficiency blue-LED-excitable NIR-II-emitting MgO:Cr^3+^, Ni^2+^ phosphor for future broadband light source toward multifunctional NIR spectroscopy applications. *Chem. Eng. J.* **452**, 139313 (2023).

[28] Shannon, R. D. Revised effective ionic radii and systematic studies of interatomie distances in halides and chaleogenides. *Acta Cryst.* **32**, 751–767 (1976).

[29] Mironova-Ulmane, N. et al. Crystal field calculations of energy levels of the Ni^2+^ ions in MgO. *J. Lumin.* **135**, 74–78 (2013).

[30] Inokuti, M. & Hirayama, F. Influence of energy transfer by the exchange mechanism on donor luminescence. *J. Chem. Phys.* **43**, 1978–1989 (1965).

[31] Satpathy, A. et al. Near‐Infrared I/II nanophosphors with Cr^3+^/Ni^2+^ energy transfer for bioimaging. *Adv. Optical Mater.* **11**, 2300321 (2023).

[32] Dexter, D. L. A theory of sensitized luminescence in solids. *J. Chem. Phys.* **21**, 836–850 (1953).

[33] Baur, W. H. The geometry of polyhedral distortions. Predictive relationships for the phosphate group. *Acta Crystallogr. Sect. B Struct. Sci.* **30**, 1195–1215 (1974).

[34] Peli, E. Contrast in complex images. *J. Opt. Soc. Am. A* **7**, 2032 (1990). 2231113 10.1364/josaa.7.002032

[35] Najjar, Y. Effect of contrast measures on the performance of no-reference image quality assessment algorithm for contrast-distorted images. *Jordan J. Elec. Eng.* **7**, 390–404 (2021).

[36] Kresse, G. et al. Efficient iterative schemes for ab initio total-energy calculations using a plane-wave basis set. *Phys. Rev. B* **54**, 11169–11186 (1996). 10.1103/physrevb.54.11169 9984901

[37] Kresse, G. et al. Efficiency of ab-initio total energy calculations for metals and semiconductors using a plane-wave basis set. *Comput. Mater. Sci.* **6**, 15–50 (1996).

[38] Perdew, J. P. et al. Generalized gradient approximation made simple. *Phys. Rev. Lett.* **77**, 3865–3868 (1996). 10062328 10.1103/PhysRevLett.77.3865

[39] Adamo, C. & Barone, V. Toward reliable density functional methods without adjustable parameters: the PBE0 model. *J. Chem. Phys.* **110**, 6158–6170 (1999).

[40] Kresse, G. & From, D. J. ultrasoft pseudopotentials to the projector augmented-wave method. *Phys. Rev. B* **59**, 1758–1775 (1999).

[41] Blöchl, P. E. Projector augmented-wave method. *Phys. Rev. B* **50**, 17953–17979 (1994). 10.1103/physrevb.50.17953 9976227

