# Supplementary material for: Laser-driven luminescent ceramic-converted near-infrared II light source for advanced imaging and detection techniques
Source: Light Sci Appl. 2025 Sep 11;14:317. doi: 10.1038/s41377-025-01953-4 (PMC12426208; doi:10.1038/s41377-025-01953-4)
Supplement: Supplementary file 1 — Suppoting information [file 41377_2025_1953_MOESM1_ESM.docx]

**Supporting information**

**Laser-driven luminescent ceramic-converted near-infrared II light source for advanced imaging and dete****ction techniques**

Simin Gu^1^, Huiwang Lian^1^, Rongyi Kuang^1^, Bibo Lou^2^, Chonggeng Ma^2*^, Gaochao Liu^3^, Jing Wang^1,4*^

^1^ Ministry of Education Key Laboratory of Bioinorganic and Synthetic Chemistry, State Key Laboratory of Optoelectronic Materials and Technologies, School of Chemistry, Sun Yat-Sen University, Guangzhou, 510275, China

^2^ School of Optoelectronic Engineering & CQUPT-BUL Innovation Institute, Chongqing University of Posts and Telecommunications, Chongqing 400065, P.R. China

^3^ The State Key Laboratory of Luminescent Materials and Devices, Guangdong Provincial Key Laboratory of Fiber Laser Materials and Applied Techniques, School of Materials Science and Technology, South China University of Technology, Guangzhou, 510641, PR China

^4^ Northeast Guangdong Key Laboratory of New Functional Materials, Guangdong Rare Earth Photofunctional Materials Engineering Technology Research Center, School of Chemistry and Environment, Jiaying University, Meizhou, 514015, P.R. China

* Corresponding authors.

E-mail address: [ceswj@mail.sysu.edu.cn](mailto:ceswj@mail.sysu.edu.cn) (J. Wang); cgma.ustc@gmail.com (C.-G Ma).


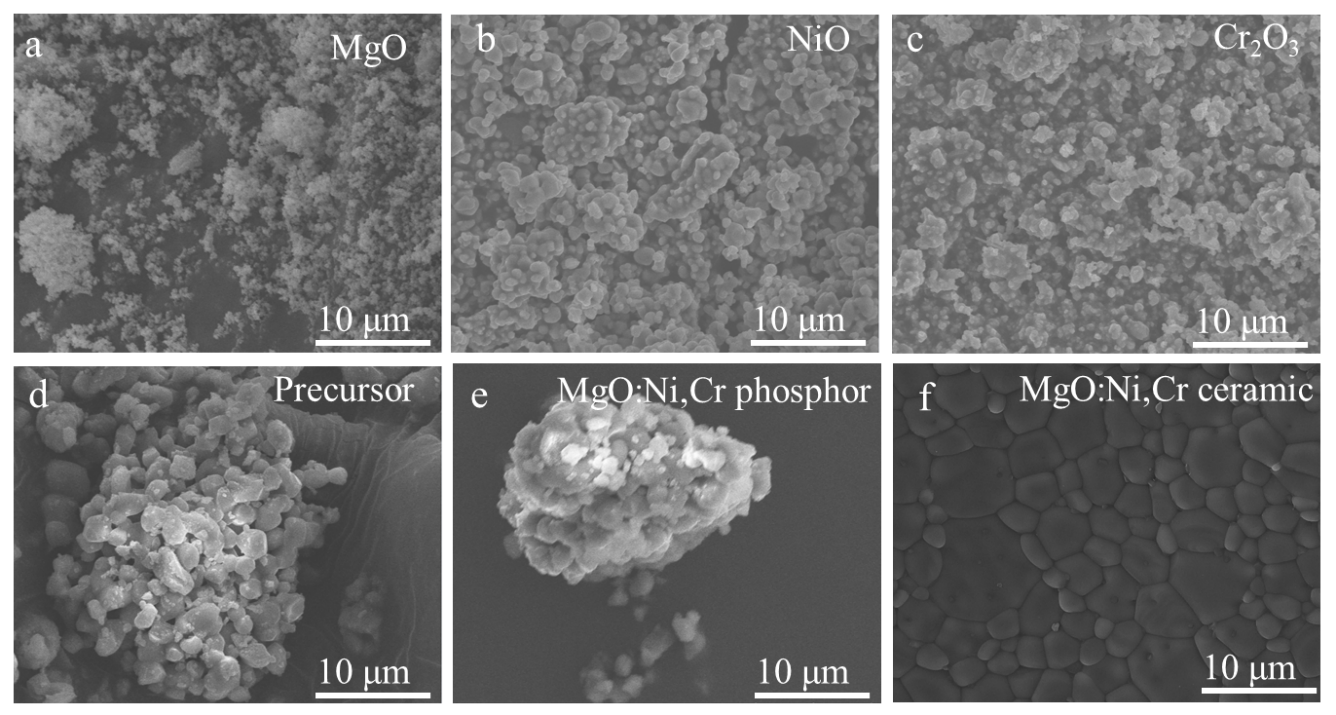


**Figure S1** SEM images of a) MgO, b) NiO, c) Cr_2_O_3_ raw materials, d) precursor powder after ball-milling, e) MgO:0.3%Ni^2+^,0.3%Cr^3+^ phosphor and f) MgO:0.3%Ni^2+^,0.3%Cr^3+^ ceramic.

**Figure S2** XRD patterns of a) MgO:*x*%Ni^2+^,0.3%Cr^3+^ (*x* = 0.1-0.7) ceramics and b) MgO:0.5%Ni^2+^,*y*%Cr^3+^ (*y* = 0.1-0.7) ceramics.


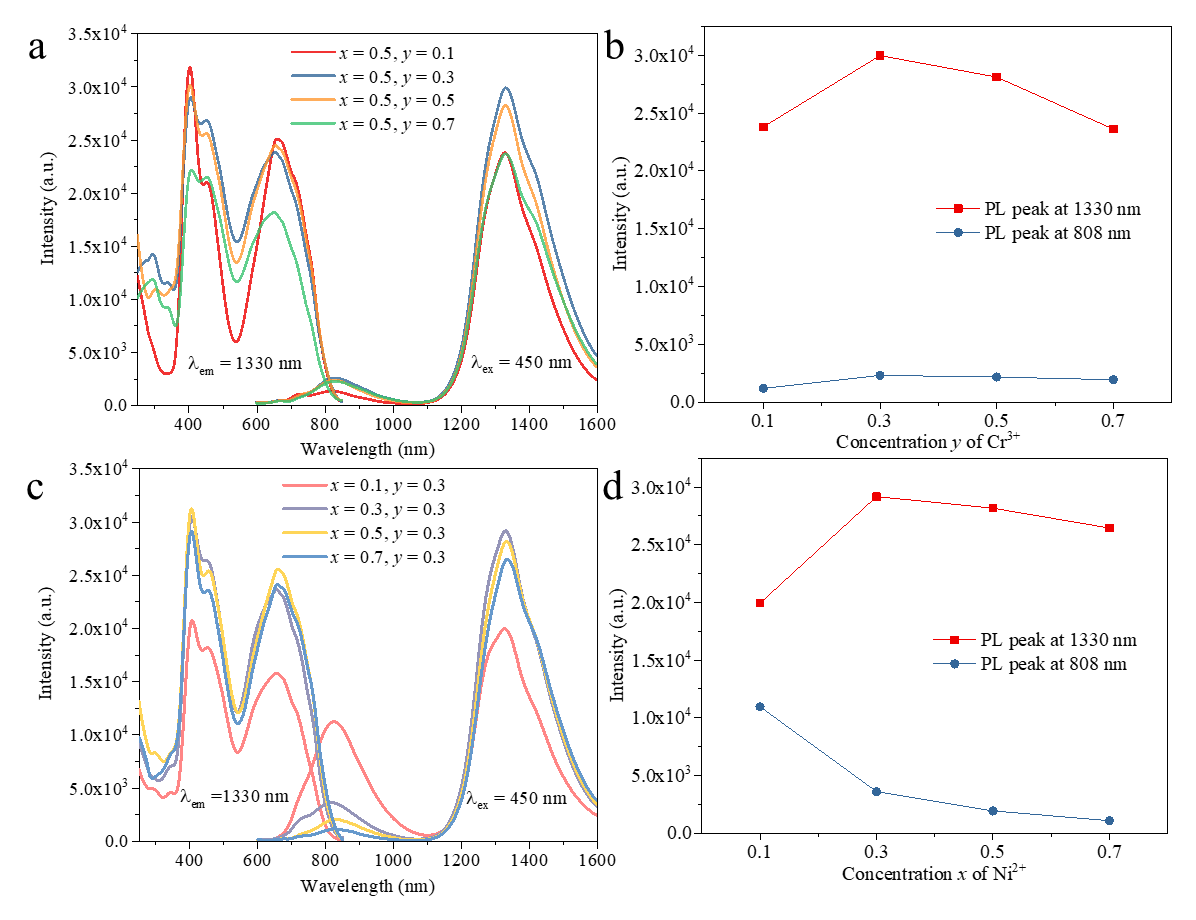


**Figure S3** a) PLE/PL spectra of MgO:0.5%Ni^2+^,*y*%Cr^3+^ (*y* = 0.1-0.7) ceramics. b) The concentration dependence of integrated NIR emission intensity of Ni^2+^ at 1330 nm and Cr^3+^ at 808 nm in MgO:0.5%Ni^2+^,*y*%Cr^3+^ (*y* = 0.1-0.7) ceramics. Both the PL intensity of Cr^3+^ and Ni^2+^ enhance as the concentration y of Cr^3+^ increases from 0.1% to 0.3%, evidencing appearance of energy transfer from Cr^3+^ to Ni^2+^ ions in low Cr^3+^ doping concentration range. c) PLE/PL spectra of MgO:*x*%Ni^2+^,0.3%Cr^3+^ (*x* = 0.1-0.7) ceramic. d) The concentration dependence of integrated NIR emission intensity of Ni^2+^ at 1330 nm and Cr^3+^ at 808 nm in MgO:*x*%Ni^2+^,0.3%Cr^3+^ (*x* = 0.1-0.7) ceramics. The optimal composition of ceramic is determined to be MgO:0.3%Ni^2+^,0.3%Cr^3+^, based on its highest NIR-II emission intensity.

**Figure S4** Decay curves of Cr^3+^ in MgO:*x*%Ni^2+^,0.3%Cr^3+^ (*x* = 0-0.7) ceramics. With the increasing concentration of acceptor Ni^2+^, the lifetime of donor Cr^3+^ shorten continuously.

**Figure S5** Quantum efficiency of MgO:*x*%Ni^2+^,0.3%Cr^3+^ (*x* = 0.1-0.7) ceramics when the excitation was fixed at 450 nm and the emission was monitored at 1000-1650 nm.

**Figure S6** Quantum efficiency of MgO:0.3%Ni^2+^,0.3%Cr^3+^ phosphor when the excitation was fixed at 450 nm and the emission was monitored at 1000-1650 nm.


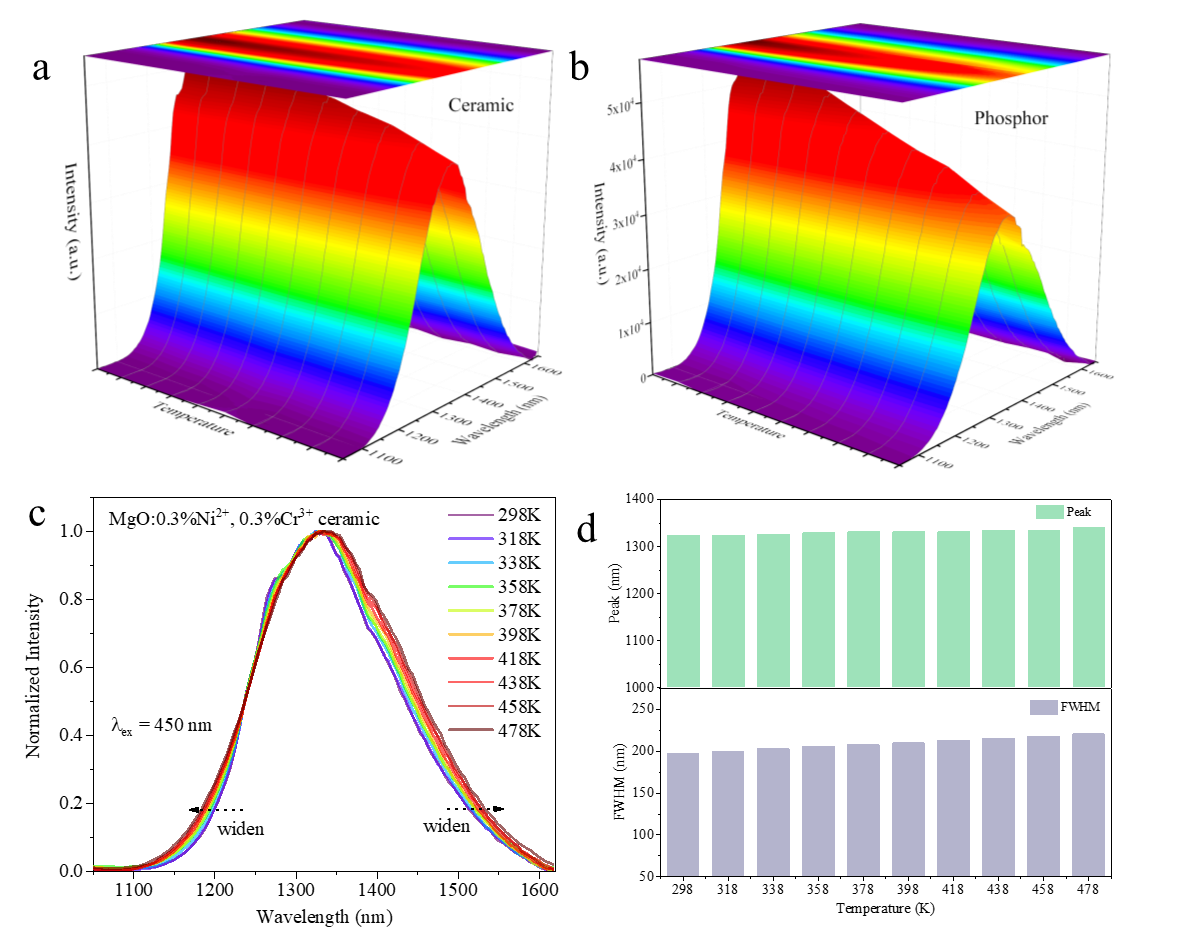


**Figure S7** Temperature-dependent PL spectra of a) MgO:0.3%Ni^2+^,0.3%Cr^3+^ ceramic and b) MgO:0.3%Ni^2+^,0.3%Cr^3+^ phosphor in the temperature ranging from 298 K to 478 K. c) Normalized PL spectra of MgO:0.3%Ni^2+^,0.3%Cr^3+^ ceramic in temperature ranging from 298 to 478 K. d) The temperature-dependence of NIR-II emission peak position and FWHM of MgO:0.3%Ni^2+^,0.3%Cr^3+^ ceramic.

**Figure S8** The specific heat capacity, density, thermal diffusivity, and thermal conductivity of blank MgO ceramic and MgO:0.3%Ni^2+^,0.3%Cr^3+^ ceramic.

**Figure S9** Detected output power of the laser-driven device based on blank MgO ceramic under different blue laser power densities. Used to correct the NIR-II output power of the as-fabricated laser-driven NIR-II lighting source.


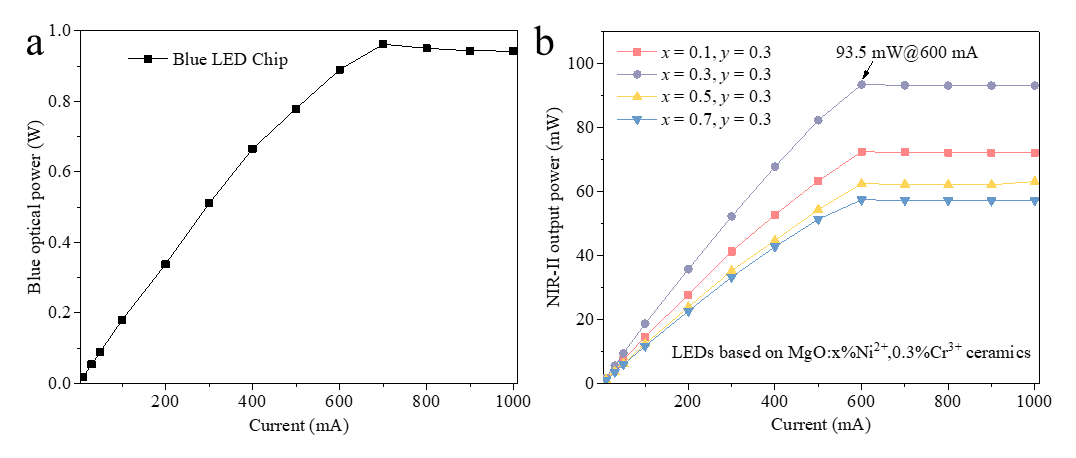


**Figure S10** a) Blue optical power of 450 nm blue LED chip under operated at different input currents. b) NIR-II output power of LED-driven NIR-II lighting sources based on MgO:*x*%Ni^2+^,0.3%Cr^3+^ (*x* = 0.1-0.7) ceramics under operated at different input currents.


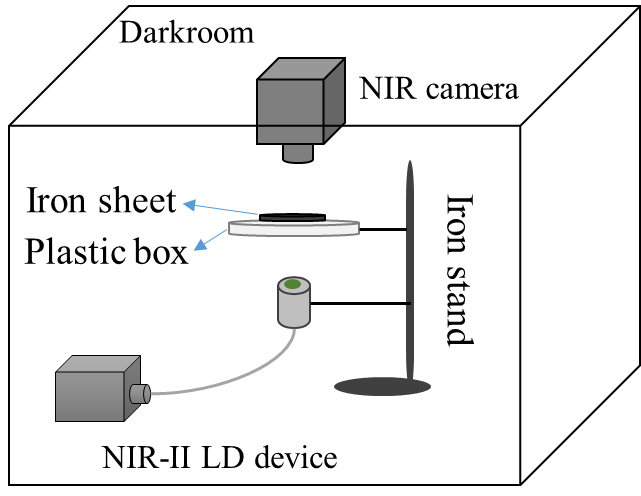


**Figure S11** Schematic diagram of NIR images capture measurement of an iron sheet on a plastic box.

Table S1 The fitting parameters of lifetime of Cr^3+^ in MgO:*x*%Ni^2+^,0.3%Cr^3+^ (*x* = 0-0.7) ceramics in Figure S4.

| Sample | *C_1_* | *τ_1_* | C_2_ | *τ_2_* | *τ(ms)* | R^2^ |
| --- | --- | --- | --- | --- | --- | --- |
| *x* = 0, *y* = 0.3 | 10596.33 | 0.09336 | 684.55 | 0.8326 | 0.364 | 0.999 |
| *x* = 0.1, *y* = 0.3 | 10539.14 | 0.07643 | 508.88 | 0.6478 | 0.242 | 0.999 |
| *x* = 0.3, *y* = 0.3 | 11060.66 | 0.04742 | 182.49 | 0.6072 | 0.145 | 0.999 |
| *x* = 0.5, *y* = 0.3 | 10082.28 | 0.0304 | 230.15 | 0.2634 | 0.069 | 0.999 |
| *x* = 0.7, *y* = 0.3 | 7806.44 | 0.01435 | 2273.17 | 0.0466 | 0.030 | 0.999 |

Table S2 The calculated average Mg-O and Ni-O bond lengths and distortion indexes of MgO_6_ and NiO_6_ octahedron.

| **Model** | **Site** | **Average bond length (Å)** | **Distortion index** |
| --- | --- | --- | --- |
| MgO | MgO_6_ | 2.128 | 0 |
| MgO:Ni^2+^ | MgO_6_ | 2.127 | 0.0020 |
|  | NiO_6_ | 2.135 | 0 |
| MgO:Ni^2+^,Cr^3+^ (Ni-Cr 3.03 Å) | NiO_6_ | 2.130 | 0.0072 |
| MgO:Ni^2+^,Cr^3+^ (Ni-Cr 4.28 Å) | NiO_6_ | 2.136 | 0.0137 |
| MgO:Ni^2+^,Cr^3+^ (Ni-Cr 5.21 Å) | NiO_6_ | 2.133 | 0.0025 |
| MgO:Ni^2+^,Cr^3+^ (Ni-Cr 6.01 Å) | NiO_6_ | 2.135 | 0.0037 |
| MgO:Ni^2+^,Cr^3+^ (Ni-Cr 6.72 Å) | NiO_6_ | 2.133 | 0.0026 |

Table S3 Details about absorption, IQE, and EQE of MgO:*x*%Ni^2+^,0.3%Cr^3+^ (*x* = 0.1-0.7) ceramics and MgO:0.3%Ni^2+^,0.3%Cr^3+^ phosphor when the excitation was fixed at 450 nm and the emission was monitored at 1000-1650 nm.

| **Sample** | **Abs** | **IQE** | **EQE** |
| --- | --- | --- | --- |
| *x* = 0.1, *y* = 0.3 | 0.642 | 39.70 | 25.49 |
| *x* = 0.3, *y* = 0.3 | 0.650 | 61.06 | 39.69 |
| *x* = 0.5, *y* = 0.3 | 0.638 | 60.62 | 38.68 |
| *x* = 0.7, *y* = 0.3 | 0.642 | 53.39 | 34.28 |
| Phosphor | 0.343 | 83.97 | 28.80 |

Table S4 Performance comparison of MgO:0.3%Ni^2+^,0.3%Cr^3+^ ceramic developed in this work and previously reported broadband NIR-II-emitting Ni^2+^-doped luminescent materials.

| Composition | Emission band (nm) | Emission peaks (nm) | IQE (%) | EQE (%) | Thermal stability | LED Device | Ref. |
| --- | --- | --- | --- | --- | --- | --- | --- |
| YAGG- Ni^2+^-H_3_BO_3_ | 1000-1700 | 1450 | 54 | 8.2 | 71% at 423 K | 1.25 mW at 300 mA | ^1^ |
| MgGa_2_O_4_:Cr^3+^,Ni^2+^ | 1000-1600 | 1260 | 96.5 | 29.4 | 67.9% at 423 K | 14.9 mW at 150 mA | ^2^ |
| MgTi_2_O_5_:Ni^2+^ | 1000−1700 | 1450 | 21.4 | 10.8 | ~20% at 375 K | 3.6 mW at 300 mA | ^3^ |
| LiMgPO_4_:Cr^3+^,Ni^2+^ | 1100-1600 | 1380 | 5.2 | ˗ | 56.6% at 293 K | 2.7 mW at 120 mA | ^4^ |
| MgO:Ni^2+^,Li^+^ | 1000-1700 | 1460 | 42.9 | 9.6 | 60.5% at 423 K | 10.1 mW at 350 mA | ^5^ |
| MgO:Cr^3+^,Ni^2+^ | 1000-1700 | 1335 | 92.7% | 25.9 | 83.0% at 423 K | 27.4 mW at 350 mA | ^6^ |
| Mg_3_Ga_2_GeO_8_:Ni^2+^ | 650−1600 | 1410 | 36.7 | 7.3 | 56.2% at 373 K | - | ^7^ |
| Ca_2_GeO_4_:Ni^2+^ | 1000-1400 | 1164 | 23.1 | 3.6 | 26.2% at 423 K | ˗ | ^8^ |
| SrTiO_3_:Ni^2+^ | 1000-1600 | 1311 | 6.5 | ˗ | ~20% at 290 K | ˗ | ^9^ |
| SrTiO_3_–Ni^2+^–Yb^3+^ | 1000-1600 | 1250 | 27.46 | ˗ | 54.46% at 423 K | ˗ | ^10^ |
| Ba_2_MgWO_6_:Ni^2+^ | 1200−2000 | 1650 | 16.67 | ˗ | 50% at 423 K | ˗ | ^11^ |
| (Zn/Mg)Ga_2_O_4_:Ni^2+^ | 1000-1600 | 1300 | ~ 12 | ˗ | ~50 % at 393 K | ˗ | ^12^ |
| LiGa_4.9_Sn_0.1_O_8_: Ni^2+^ | 1000-1650 | 1300 | 20.6 | ˗ | ˗ | ˗ | ^13^ |
| ZnGa_2_O_4_:Ni^2+^, Eu^3+^ | 1000-1650 | 1310 | ˗ | ˗ | 53.4% at 423 K | ˗ | ^14^ |
| LiGa_5_O_8_: Ni^2+^ Glass-Ceramic | 1100-1600 | 1300 | 10±2 | ˗ | ˗ | ˗ | ^15^ |
| MgO:0.3%Ni^2+^,0.3%Cr^3+^ Ceramic | 1000-1650 | 1330 | 61.06 | 39.69 | 92.11% at 478 K | 93.2 mW at 600mA  (LED device)  214 mW at 21.43 W·mm^-2^ (LD device) | Our work |

$D_{\mathrm{dis}}=\frac{1}{n}\sum_{i=1}^{n} \frac{\left| d_{i}-\left. d_{av} \right| \right.}{d_{av}}$ (1)

Equation S1 The calculation formula of distortion index of MgO_6_ and NiO_6_ octahedron in MgO , MgO:Ni^2+^ and MgO:Ni^2+^,Cr^3+^. Where D*_dis_* is the lattice distortion, and n is the coordination number. d*_i_* is the distance from Mg or Ni to the i-th coordinating O atoms, and d*_av_* is the average bond length ^16^. The specific fitting results were listed in Table S1.

$I(t)=I_{0}+C_{1}\exp\left( \frac{-t}{\tau_{1}} \right)+C_{2}\exp\left( \frac{-t}{\tau_{2}} \right)$ (2-1)

$\tau=\frac{C_{1}\tau_{1}^{2}+C_{2}\tau_{2}^{2}}{C_{1}\tau_{1}+C_{2}\tau_{2}}$ (2-2)

Equation S2 The fitting formula of lifetime of MgO:*x*%Ni^2+^,0.3%Cr^3+^ ceramics (*x* = 0-0.7) in Supplementary Fig. 4. Where *I* and *I_0_* in (2-1) stand for the photoluminescence intensity at time *t* and initial, *C_1_* and *C_2_* refer to pre-exponential factors, *τ_1_* and *τ_2_* are the lifetime components ^17,18^, and *τ* in (2-2) is the average lifetime ^19^. The specific fitting results were listed in the inset of Figure S4.

$\eta_{ET}=1- \frac{\tau}{\tau_{0}}$ (3)

Equation S3 The calculation equation of energy transfer efficiency of MgO:*x*%Ni^2+^,0.3%Cr^3+^ ceramics (*x* = 0.1-0.7) ^2^. Where *τ* and *τ*_0_ refer to the average lifetime of Cr^3+^ at 808 nm emission in the presence and absence of Ni^2+^.

$\eta_{IQE}=\frac{\int L_{S}}{\int E_{R}-\int E_{S}}$ (4-1)

$\eta_{EQE}=\frac{\int L_{S}}{\int E_{R}}$ (4-2)

Equation S4 The calculation formula of quantum efficiency of MgO:*x*%Ni^2+^,0.3%Cr^3+^ ceramics (*x* = 0.1-0.7) and MgO:0.3%Ni^2+^,0.3%Cr^3+^ phosphor. Herein, *η*_IQE_ in (4-1) and *η*_EQE_ in (4-2) are the internal and external quantum efficiency, where *L_S_* stands for the emissive photons of the sample, *E_R_* and *E_S_* are the reflected photons of excitation light with the blank reference and sample ^20^, respectively. The specific fitting results were listed in Figure 2d and 2f, Figure S5, Fig. S6 and Table S2, Table S3.

$\lambda=\rho\times c\timesɑ$ (5)

Equation S5 The calculation formula of thermal conductivity λ, where ρ, с and ɑ are the density, specific heat capacity and thermal diffusivity, respectively ^21^. The detailed parameters were listed in Figure S8.

$C=\frac{L_{W}-L_{B}}{L_{W}}$ (6)

Equation S6 The contrast estimated by Weber Contrast Equation^22,23^, where *C* is the contrast, *L_w_* is the average gray of the white-ring of the plastic box, *L_B_* is the average gray of the black-ring of the iron sheet.

**Supplementary Method**

**Characterization**

The thermal conductivity was measured through laser flash method. The test temperature was 298 K. Density is obtained by dividing mass by volume and mass and volume were measured by Electronic balance (BSA224S-CW) and Digital thickness meter, respectively. Specific heat capacity was measured by Differential scanning calorimeter (DSC 204F1) and thermal diffusivity was measured by Laser thermal conductivity meter (LFA467).

**Supplementary References**

[1] Yuan, L. et al. Ni^2+^-doped garnet solid-solution phosphor-converted broadband shortwave infrared light-emitting diodes toward spectroscopy application. *ACS Appl. Mater. Interfaces* **14**, 4265-4275, (2022).

[2] Miao, S. et al. Broadband short-wave infrared-emitting MgGa_2_O_4_:Cr^3+^, Ni^2+^ phosphor with near-unity internal quantum efficiency and high thermal stability for light-emitting diode applications. *ACS Appl. Mater. Interfaces* **15**, 32580-32588, (2023).

[3] Tang, C. et al. Ni^2+^-activated MgTi_2_O_5_ with broadband emission beyond 1200 nm for NIR-II light source applications. *J. Mater. Chem. C* **10**, 18234-18240, (2022).

[4] Miao, S. et al. Blue LED‐Pumped broadband short‐wave infrared emitter based on LiMgPO_4_:Cr^3+^,Ni^2+^ phosphor. *Adv. Mater. Technol.* **7**, 2200320, (2022).

[5] Liu, B. et al. Ultra-broadband and high-efficiency phosphors to brighten NIR-II light source applications. *Cell Rep. Phys. Sci.* **3**, 101078, (2022).

[6] Liu, B. et al. A High-efficiency blue-LED-excitable NIR-II-emitting MgO:Cr^3+^,Ni^2+^ phosphor for future broadband light source toward multifunctional NIR spectroscopy applications. *Chem. Eng. J.* **452**, 139313, (2023).

[7] Wang, C. et al. Efficient ultra-broadband NIR-II emission achieved by multi-site occupancy in Mg_3_Ga_2_GeO_8_: Ni^2+^ phosphor. *J. Alloy. Comp.* **942**, 168893, (2023).

[8] Huang, W. et al. Sharp-line near-infrared emission from tetrahedron-occupied Ni^2+^ in Ca_2_GeO_4_. *Inorg. Chem.* **62**, 13370-13377, (2023).

[9] Gao, Y., Wang, B., Liu, L. & Shinozaki, K. Near-infrared engineering for broad-band wavelength-tunable in biological window of NIR-Ⅱ and -Ⅲ: A solid solution phosphor of Sr_1-x_Ca_x_TiO_3_:Ni^2+^. *J. Lumin.* **238**, 118235, (2021).

[10] Zhu, F., Gao, Y., Ding, J. & Qiu, J. Synergistic enhancement of the near-infrared luminescence properties of Ni^2+^-doped SrTiO_3_ perovskite phosphors and their application. *J. Mater. Chem. C* **11**, 10236-10246, (2023).

[11] Lu, X., Gao, Y., Chen, J., Tan, M. & Qiu, J. Long-wavelength near-infrared divalent nickel-activated double-perovskite Ba_2_MgWO_6_ phosphor as imaging for human fingers. *ACS Appl. Mater. Interfaces* **15**, 39472-39479, (2023).

[12] Chen, J., Gao, Y., Tan, M., Lu, X. & Qiu, J. Cation substitution-dependent phase transforming phosphors: A new alternative broadband NIR-II emitter for solid state lighting. *J. Alloy. Comp.*  **965**, 171311, (2023).

[13] Kosgei, G. K. et al. Exploring the luminescence of tin‐incorporated nickel‐sensitized lithium gallate short‐wave‐infrared phosphors. *J. Am. Ceram. Soc.* **106**, 6342-6351, (2023).

[14] Jin, M., Zhang, T., Li, J. G. & Zhu, Q. Incorporation of Eu^3+^ in ZnGa_2_O_4_:Ni^2+^ for improved NIR persistent luminescence located in second transparency window. *J. Am. Ceram. Soc.* **107**, 265-275, (2023).

[15] Suzuki, T., Arai, Y. & Ohishi, Y. Quantum efficiencies of near-infrared emission from Ni^2+^-doped glass-ceramics. *J. Lumin.* **128**, 603-609, (2008).

[16] Yang, Z. et al. Giant red-shifted emission in (Sr,Ba)Y_2_O_4_:Eu^2+^ phosphor toward broadband near-infrared luminescence. *Adv. Funct. Mater.* **32**, 2103927, (2022).

[17] Zhong, Y. et al. Enhancing quantum efficiency and tuning photoluminescence properties in far-red-emitting phosphor Ca_14_Ga_10_Zn_6_O_35_:Mn^4+^ based on chemical unit engineering. *Chem. Eng. J.* **374**, 381-391, (2019).

[18] Wang, X. et al. Fabrication of a wide color gamut pc-WLED surpassing 107% NTSC based on a robust luminescent uranyl phosphate. *Chem. Mater.* **33**, 6329-6337, (2021).

[19] Zhou, J. & Xia, Z. Luminescence color tuning of Ce^3+^, Tb^3+^ and Eu^3+^ codoped and tri-doped BaY_2_Si_3_O_10_ phosphors via energy transfer. *J. Mater. Chem. C* **3**, 7552-7560, (2015).

[20] Zhang, J. et al. Composition Screening in Blue-Emitting Li_4_Sr_1+x_Ca_0.97-x_(SiO_4_)_2_:Ce^3+^ Phosphors for High Quantum Efficiency and Thermally Stable Photoluminescence. *ACS Appl. Mater. Interfaces* **9**, 30746-30754, (2017).

[21] Kubiś, M. et al. On the anisotropy of thermal conductivity in ceramic bricks. *J. Build. Eng.* **31**, 101418, (2020).

[22] Peli, E. Contrast in complex images. *J. Opt. Soc. Am. A* **7**, 2032 (1990).

[23] Najjar, Y. Effect of contrast measures on the performance of no-reference image quality assessment algorithm for contrast-distorted images. *Jordan J. Elec. Eng.* **7**, 390-404, (2021).
